# Editorial: Experimental and theoretical investigation of non-covalent interactions in potential bioactive compounds

**DOI:** 10.3389/fchem.2023.1326955

**Published:** 2023-11-13

**Authors:** Subbiah Thamotharan, M. Judith Percino, Diego Mauricio Gil

**Affiliations:** ^1^ Biomolecular Crystallography Laboratory and DBT-Bioinformatics Center, School of Chemical and Biotechnology, SASTRA Deemed University, Thanjavur, India; ^2^ Unidad de Polímeros y Electrónica Orgánica, Instituto de Ciencias, Benemérita Universidad Autónoma de Puebla, Puebla, México; ^3^ INBIOFAL (CONICET-UNT), Instituto de Química Orgánica, Facultad de Bioquímica, Química y Farmacia, Universidad Nacional de Tucumán, San Miguel de Tucumán, Argentina

**Keywords:** weak noncovalent interaction, energetics, bioactive, DFT, CLP-PIXEL energy, hirshfeld surface

## Introduction

The properties of the bioactive solids are primarily dependent on the various types of noncovalent interactions such as hydrogen, halogen, chalcogen, pnictogen, tetrel, triel, π-stacking, cation-π and anion-π, and lone pair-π interactions (Jena et al., 2022; Schneider, 2022). Among various non-covalent interactions, hydrogen bonds and π-stacking interactions are well recognized in structural chemistry, biology, and crystal engineering. The other mentioned interactions play an essential role in synthesis, catalysis, drug design and materials science, crystal engineering, etc. either alone or in combination with multiple types of noncovalent interactions. Therefore, understanding the presence, hierarchy of interactions, energetics, and importance of different interactions is vital to understanding the biological action of compounds and designing more specific and active molecules. Several modern theoretical tools such as the Hirshfeld surface, CLP-PIXEL energy, quantum theory of atoms in molecules (QTAIM), and molecular docking aid in understanding the role of various intermolecular interactions in the context of supramolecular assemblies and biological action of crystalline solids.

In this Research Topic, we collected four research articles which highlight the importance of various weak noncovalent interactions in different systems. Saeed et al. described the crystal structure analysis of *N*-((4-acetylphenyl) carbamothioyl) pivalamide, and the molecular conformation of this molecule is locked by an intramolecular N–H⋅⋅⋅O hydrogen bond that generatesa S (6) ring Saeed et al. The intermolecular N–H⋅⋅⋅O=C and C–H⋅⋅⋅π in addition to π-stacking interactions are formed in the solid state to stabilize the crystal structure. The molecular docking analysis of this compound against four biological targets, namely, α-amylase, urease, acetylcholinesterase (AChE), and butylcholinesterase (BChE), suggests that AChE and BChE have a relatively stronger binding to the compound than the other two protein targets. These findings are in good agreement with those from the *in vitro* experiment. Gunasekaran et al. identified potential bioactive phytochemicals from leaf extracts of *Aegle marmelos*. In particular, three phytochemicals, aegeline, marmin, and marminol, showed inhibition of sperm hyaluronidase activity, as evident from the *in vitro* assay Gunasekaran et al.. Further, *in silico* molecular docking analysis reveals the key active site residues are involved in the molecular recognition of aegeline, marmin and marmenol phytochemicals. These phytochemicals establish hydrogen bonds and hydrophobic interactions at the active site residues. The topological properties of the intermolecular interactions formed between the ligands and active site residues provided the strength of these interactions. Further, the noncovalent interaction (NCI) plot reveals the nature of interactions between ligand and enzymes.

Percino et al. synthesized two positional isomers of acrylonitrile derivatives, and X-ray diffraction analysis revealed that they exhibit similar crystal packing and hence similar optical properties Percino et al.. Hirshfeld surface analysis reveals the effect of positional isomers on the intermolecular interactions formed in the crystal structures. The characteristic intramolecular C–H⋅⋅⋅C(π) interaction is observed in these structure and helps to maintain the molecular conformation. Weak C–H⋅⋅⋅π and C–H⋅⋅⋅N interactions dominate the crystal packing. The energetics of molecular dimers held by various interactions and each of these interactions was further characterized by the QTAIM approach. This study clearly demonstrated the isomeric effect using X-ray structures, and various theoretical calculations help to design novel molecules with desired properties. Aguiar et al. demonstrated the effect of water molecules on paraquat salts. DFT calculations were carried out to obtain the chemical reactivity of the bipyridylium cation, and supramolecular arrangements were analyzed Aguiar et al.. The supramolecular assemblies are generated via various short intermolecular contacts such as O–H⋅⋅⋅O and C–H⋅⋅⋅O/Cl hydrogen bonds. It is interesting to note that the energy associated with C–H⋅⋅⋅Cl is remarkably different in hydrated and anhydrous systems of paraquat salts.

In conclusion, the study of various weak molecular interactions in different molecular solids and bioactive compounds with their biological targets has gained great attention in recent years to help us understand the molecular behaviors in a solid state and in the biological system. This Research Topic clearly captures the importance of studying noncovalent interactions to better understand the system to design novel molecules with desired properties to some extent.
